# Symptoms, Prognosis, and Treatment, of Internal Hemorrhage after Delivery of the Child and Expulsion of the Placenta

**Published:** 1817-12

**Authors:** Thomas Atkinson


*For the London Medical and Physical Journal.*

### *Symptoms, Prognosis, and Treatment, of Internal Hemorrhage after Delivery of the Child and Expulsion of the Placenta.* By THOMAS ATKINSON, Esq.

IN the different Treatises on Midwifery which have passed under my inspection, I do not recollect to have ever read any observations on *internal hemorrhage*, which sometimes occurs after delivery. In these cases, the danger is great, and (no blood appearing externally) the symptoms are so very insidious, that, to those unacquainted with their true source, they may be confidently referred to causes of a transient and less alarming nature, viz. faintness, weakness consequent to labour, &c.; from such unfortunate error, the principal, and almost only, means of saving the patient would be certainly neglected, and the sacrifice of her life the probable consequence.

We not unfrequently hear of death soon after delivery which could not be satisfactorily accounted for, as every thing seemed going on well previous to the fatal event; and it is a query with myself, whether not a few of such melancholy occurrences may not be fairly referable to flooding internally; as during my own practice I have met with cases, which, from the attendant symptoms and examination, evidently arose from this insidious cause. The adoption of decisive treatment was attended with a favourable result. In one case, the patient, from the great exhaustion, was for some time in a doubtful state, but recovered. But it is unnecessary for me to add more on this subject, so clearly explained in the following lecture of Professor Hamilton, as transcribed from my note-book, and now sent for insertion in your widely circulated Journal.

"After delivery, the blood has a peculiar tendency to coagulate, from this the uterus and vagina become stopped with coagula, none appearing externally; hence it is called internal hemorrhage, or flooding, in which the danger is great; the prognosis, in consequence, must be guarded.

"We judge of the presence of internal hemorrhage by the symptoms; if the patient complains of the room turning round and of singing in the ears, we then fear hemorrhage is going on internally; if sickness and retching ensue, we are then more confirmed in our opinion; and, if the pulse become feeble, and faintings or delirium ensue, we are then sure of it. When we suspect this, we must lose no time, but introduce the fingers into the vagina, when, should hæmorrhage have taken place, we feel the vagina dilated, and plunge our fingers into coagula,—if no flooding have taken place, we feel the vagina contracted.

"The symptoms of hemorrhage internally are feigned by irregular hysteria, therefore we should never lose ourselves in doubt, but immediately examine; when its presence is ascertained, no time should be lost, the hand must be introduced into the vagina, not only to extract the coagula, but also to make pressure on the uterus, which seems as flabby as wet paper; and this must be continued till the uterus be made to contract, and, as it were, expel the hand. This practice is certainly horrible, and the woman, though in a faint, often groans during the operation; it is, however, the only one that must or can be followed, and by it many a valuable mother has been saved, who would certainly soon have died, were we to stand supinely looking on and doing nothing.

"Although this practice seems very severe, yet, in some cases, we are obliged to resort to means equally distressing. When the hand has removed the coagula, and pressed on the sides of the uterus for some time, without its contracting, then the hand may be withdrawn, and a pitcher of cold water must be dashed on the naked abdomen of the patient, without regard to decency, or the impertinence of attendants; or this may be done while the hand remains *in utero*, and a cold water glyster may be also administered. After the hæmorrhage has ceased, and the uterus contracted, it has again come on, and the uterus again been required to be dilated to the size before delivery; a third time it has been requisite to introduce the hand and cause contraction, yet the patient has recovered.

"In some cases, before introducing the hand, we give the patient a lump of sugar moistened with spirit, to support the *vis vitæ*, which it is always necessary to do also after cessation of the hemorrhage, by a large dose of opium, and this frequently repeated. In these cases, the woman must not be left for three or four hours after delivery, lest immediate death, from recurrence of the hemorrhage, be the consequence. A woman, having had flooding in former labours, should not be left until three hours after delivery. In some cases, when the hemorrhage has not been considerable, coagula may be formed in the uterus not bigger than one's fist, and no bad symptoms then ensue; but, in eight or ten days, the vital powers become enfeebled, and either death or lingering debility ensues. The consequences of leaving coagula in the uterus arise from their putrescency, similar to those of the placenta being left. A bandage round the abdomen is proper always, whether hemorrhage supervene to delivery or not. A woman, who has been much debilitated by hemorrhage, must not be allowed to suckle the infant, as marasmus is often the consequence."

*Kilham, Yorkshire; Nov.* 10, 1817.

---

*** Though the above remarks are not entirely new, yet, as we are assured the case has not occurred to, or been suspected by, some medical practitioners, we have inserted the above paper. It is impossible we should be too much alive to this important period of of the most interesting part of medicine. At the same time, where no unfavourable symptoms exist, we have not those horrors of putrefaction from the remains of the placenta which we were once taught to apprehend. On this subject, we shall gladly receive the opinions of the most experienced.

---

### COLLECTANEA MEDICA,

